# A New Rapidly Growing Dome-Shaped Choroidal Lesion in an Eye with Treated Retinoblastoma

**DOI:** 10.1155/2021/6678779

**Published:** 2021-06-14

**Authors:** Babak Masoomian, Maria Pefkianaki, Fariba Ghassemi, Hamid Riazi-Esfahani

**Affiliations:** ^1^Ocular Oncology Service, Farabi Eye Hospital, Tehran University of Medical Sciences, Tehran, Iran; ^2^Ocular Oncology Service, OMMA Eye Center, Hygeia Hospital, Athens, Greece

## Abstract

**Background:**

To describe an extensive untreatable choroidal metastasis by retinoblastoma in the treated patient which was clinically indistinguishable from regular tumor recurrence.

**Methods:**

A 24-month-old girl without a family history of retinoblastoma (RB) was discovered to have group C RB in her right eye and group D in her left eye. The patient received 12 cycles of intravenous chemotherapy, intra-arterial chemotherapy (IAC), and intravitreal chemotherapy for the left eye and focal adjuvant therapy (laser thermotherapy and cryotherapy) for both eyes. Six months after the last treatment, fundus examination showed a regressed tumor in both eyes. Ten months after the last treatment, except for in addition to tumor recurrence, rising intraocular pressure was noticed in the left eye. While doing IAC for the left eye, a very rapid growing yellowish dome-shaped mass was found which had doubled in size in two weeks. Enucleation was considered for her.

**Results:**

Pathology evaluation of the enucleated eye revealed a very massive dome-shaped choroidal metastasis invasion with poorly differentiated RB tumor. Prophylactic systemic chemotherapy was performed for the patient.

**Conclusion:**

Choroidal metastasis in RB patients is often diagnosed based on pathology reports, but it may rarely be seen in clinical examinations especially if the pattern of tumor recurrence and growth is abnormal.

## 1. Introduction

Retinoblastoma (RB) is a highly malignant intraocular tumor, but due to new treatment options, therapy for this malignancy has evolved from enucleation and sometimes fatal disease to more conservative therapy, often with globe salvage [1]. When tumors are localized to the retina, in the presence of new treatment modalities, a good prognosis can often be predicted for patients, whereas in aggressive types such as the presence of anterior segment involvement, iris neovascularization with rising intraocular pressure (IOP), and diffuse-type tumor, enucleation seems a reasonable treatment modality [1].

Diffuse choroidal invasion is one of the bad prognostic factors for retinoblastoma cases that are mostly diagnosed after pathological examination, and it has rarely been identified with ophthalmoscopy or other clinical examination [2, 3]. Unidentified massive choroidal invasion with RB is an unpleasant event accompanied by the rapid growth of the tumor and will minimize the chance of saving the eye [2–4]. Herein, we report a 4-year-old girl who had been treated for bilateral RB, in which within 2 months a large incurable dome-shaped choroid-ciliary ciliochoroidal mass was formed in her left eye. Histopathology examination after enucleation revealed massive dome-shaped choroidal invasion by retinoblastoma cells.

## 2. Case Report

A 24-month-old girl without a family history of RB was diagnosed with bilateral retinoblastoma, group C in the right eye (OD) and group D in her left eye (OS) based on the International Classification of Retinoblastoma.

**Figure 1:**
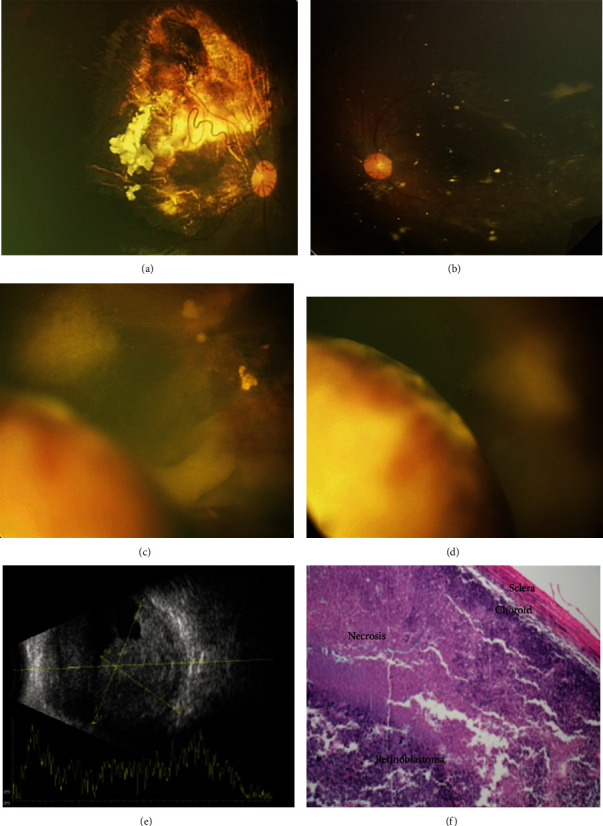
Fundus photograph of the right eye after 16 sessions of systemic chemotherapy demonstrating significant response to treatment (a). In the left eye, fundus photograph shows multiple chorioretinal scars with scattered calcified vitreous seeds (b). Four months later, fundus pictures revealed a dome-shaped yellowish mass with overlying serous retinal detachment in the left eye (c). Two weeks later, there was significant lesion growth (d). Left eye B-scan ultrasonography reveals a huge underlying choroidal mass measuring 20 × 14 mm without any highly reflective foci (e). Photomicrograph of globe lesion, in lower magnification, shows massive choroidal invasion invading the whole thickness of the choroid which extended to the sclera (hematoxylin-eosin, ×40) (f).

The patient endured 2 separate courses of systemic chemotherapy using vincristine (1.5-2 mg/m^2^ body surface area), etoposide (150 mg/m^2^ body surface area), and carboplatin (550-600 mg/m^2^ body surface area) 12 times in total, 1 course of intra-arterial chemotherapy (IAC) using Melphalan (5 mg) and Topotecan (2 mg) for the left eye, and 3 courses of intravitreal chemotherapy using Melphalan (25 *μ*g) and Topotecan (30 *μ*g) for the left eye as well as focal adjuvant therapy (laser thermotherapy and cryotherapy) for both eyes. At 4 years of age, 6 months after the last treatment, fundus examination showed a regressed tumor in both eyes without any signs of activity (Figures 1(a) and 1(b)). Ten months after the last treatment, indirect ophthalmoscopy revealed multiple recurrent lesions in the inferior quadrant of the left eye anterior to the equator near the ciliary body. The decision was made to treat the patient with IAC of Melphalan (5 mg) and Topotecan (2 mg). She underwent 2 cycles of monthly IAC adjunctive with triple freeze-thaw technique cryotherapy. The patient had intraocular pressure (IOP) rising, so topical eye drops (timolol 0.5%) were prescribed for her left eye. One month after the last IAC, surprisingly, we found a big inferonasal yellowish mass and adjacent retinal detachment with scant vitreous hemorrhage on it (Figure 1(c)). Under close observation, 2 weeks later, despite our decision for doing Ru106 plaque radiotherapy, indirect ophthalmoscopy showed rapidly growing ciliary body mass (Figure 1(d)). Ultrasound biomicroscopy showed a huge intraocular lesion with moderate internal reflectivity without any solid mass or remarkable calcification or highly reflective foci involving the choroid/ciliary body with dimensions of 20 × 14 mm (Figure 1(e)). With slit-lamp examination, the yellowish mass was visible exactly behind the lens posterior capsule. So we had a rapidly growing dome-shaped intraocular lesion whose nature was clinically unknown for us until the pathology determined its nature. The clinical view of the lesion was not similar to the classic recurrence of RB; therefore, due to high IOP, retinal detachment, and unresponsive very rapid growing, enucleation was considered for her. Due to rapidly growing and extensive tumor recurrence, resistance to treatment, and raising IOP, we decided to do enucleation.

Histopathology examination demonstrated extensive involvement of choroid, ciliary body, and angle with poorly differentiated retinoblastoma cells (Figure 1(f)). There was no involvement of optic nerve and scleral tissue. After enucleation, due to extensive choroidal involvement, 6 additional courses of systemic chemotherapy (for prophylaxis) were recommended. One year after enucleation, there were no metastases elsewhere; also, in the right eye, the regressed RB tumor was stable without any recurrence.

## 3. Discussion

Choroidal invasion by retinoblastoma is commonly seen through enucleation and pathological examination, but it has rarely been identified and reported during indirect ophthalmoscopy [2, 3].

Based on histopathology reports, massive choroidal invasion, defined according to the International Retinoblastoma Staging Working Group, is a lesion with a diameter of ≥3 mm in thickness and width [5]. The retinal pigment epithelium (RPE) and Bruch's membrane complex seem to demonstrate a unique resistance to this neoplasm, but this resistance may be disrupted in the presence of high IOP as well as in exophytic types of RB [2, 3]. The exact mechanism is unclear, but the high pressure may somehow force tumor cells and cause a breakdown in the retinal pigment epithelium/Bruch's membrane complex and allow access of RB cells to the underlying tissues [2, 3]. According to Shields et al.'s study on 289 enucleated eyes with retinoblastoma, the clinical factors that significantly predict the choroidal invasion of retinoblastoma were increased IOP and iris neovascularization. Also, the main histopathological factor that predicts choroidal invasion was a poorly differentiated tumor [3]. Our patient had two predisposition factors for aggressive choroidal invasion according to Shields et al.'s study, including high IOP and pathologically aggressive tumor [3].

Massive choroidal invasion in dome-shaped configuration is not a common finding, and there are few reports about it [2–4]. It seems that new treatment modalities increase the chance of saving the eye in RB cases, which increases the likelihood of seeing this clinical picture. Choroidal invasion of retinoblastoma is an important risk factor for metastases [3, 6–8]. According to Bosaleh et al.'s report, of the 164 enucleated eyes with isolated choroidal invasion, patients with massive choroidal invasion (>3 mm) had a higher risk of recurrence compared to those with focal invasion [8]. As mentioned before, detection of choroidal involvement with RB by indirect ophthalmoscopy in early stages is not easy, so simultaneous usage of ultrasound biomicroscopy (for detecting ciliary body mass) in follow-up visits may be helpful for early diagnosis.

Based on our knowledge, except in 2 published case reports [2, 9], saving the eyes were not feasible for patients with clinically diagnosed choroidal invasion; therefore, enucleation had been inevitable [3, 4, 6, 7]. In our patient, after observing a recurrence in the area close to the ciliary body, the patient had undergone two unsuccessful courses of IAC. In comparison to choroidal mass, ciliary body RB does not respond to IAC very well presumably due to its poor vascularization [10].

In summary, we described a mystery RB patient with a massive dome-shaped choroidal invasion. The sudden appearance of an extraordinary rapidly growing mass in undergoing treatment RB patients should make suspect a possible extensive involvement of choroid tissue (especially in cases with high IOP). However, it should be kept in mind that enucleation should be recommended as a conservative treatment in order not to jeopardize survival if prompt management and close follow-up of an RB tumor with choroidal invasion are not feasible.

## Data Availability

The datasets used in the current study are available upon reasonable request.

## Abbreviations

RB: Retinoblastoma
IAC: Intra-arterial chemotherapy
RPE: Retinal pigment epithelium.
